# Positive mental wellbeing or symptoms of depression? Discriminant validity of the Warwick-Edinburgh Mental Wellbeing Scale

**DOI:** 10.1186/s12888-025-06922-0

**Published:** 2025-05-15

**Authors:** Leif Edvard Aarø, Marit Knapstad, Otto Robert Smith

**Affiliations:** 1 Department of Health Promotion, Norwegian Institute of Public Health, Zander Kaaes Gt. 7, Bergen, NO-5015 Norway; 2 Centre for Evaluation of Public Health Measures, Norwegian Institute of Public Health, PO Box 4404, Nydalen, NO-0403 Norway; 3 Department of Teacher Education, NLA University College, PO Box 74, Sandviken, NO-5812 Norway

**Keywords:** PHQ-9, WEMWBS, Discriminant validity, Bifactor modelling, Survey instruments, Depression, Wellbeing

## Abstract

**Background:**

Scales for the measurement of mental wellbeing and psychological distress are often used as if they measure different underlying concepts. This assumption is addressed in the present study by examining the discriminant validity of the Warwick-Edinburgh Mental Wellbeing Scale (WEMWBS) with respect to the Patient Health Questionnaire (PHQ-9).

**Methods:**

The present study is based on data (n = 1690) from a baseline data collection which was carried out as part of the evaluation of ‘Prompt Mental Health Care’ (PMHC), the Norwegian Version of the British ‘Improving Access to Psychological Therapies’ (IAPT) services. PMHC offers low-threshold treatment for patients with mild to moderate depression or anxiety. Three out of four of the sample were women and three out of four were in the age range 21–50 years. Data were examined by means of structural equation- and latent variable modeling, including bifactor analysis and MIMIC models. Both the 7-item and 14-item versions of the WEMWBS were considered.

**Results:**

(i) The correlations between PHQ-9 and the WEMWBS scales were strong and negative, approaching -0.80 in the latent model analyses with the full (14 items) WEMWBS scale. (ii) Psychometric indices derived from the bifactor models suggested that the WEMWBS-7 and PHQ-9 jointly, and the WEMWBS-14 and PHQ-9 jointly were essentially unidimensional. (iii) The associations between PHQ-9 and a set of demographic variables were similar to associations between the WEMWBS scales and the same set of demographic variables, only with reversed signs. (iv) Associations between the residual WEMWBS scales and a set of demographic variables decreased strongly when removing the reliable variance accounted for by the general depressive symptoms factor.

**Conclusion:**

The results of our study suggest that the WEMWBS may lack discriminant validity with regard to the PHQ-9 in a sample of primary care patients with mild-to-moderate anxiety and/or depression.

**Supplementary Information:**

The online version contains supplementary material available at 10.1186/s12888-025-06922-0.

## Background

Measurement redundancy (having multiple measurements of the same construct) between scales measuring psychological distress and scales measuring mental wellbeing has received some attention, but there are relatively few studies that have sufficiently examined this problem [1–4]. Redundancy between scales is acknowledged as a general problem in psychology as new instruments are steadily developed without the overlap with already existing instruments being adequately considered [5]. Excessive overlap between scales may not only lead to unnecessary lengthy questionnaires, but also to misleading inferences with regard to associations and predictions [2]. As explained in a recent publication by Hays and Fayers, overlap between scales may lead to tautological inferences about the “impact” of the former on the latter, inaccurate conclusions regarding the prognostic value of these measures on relevant outcomes, and a decrease in the possibility of these scales to have unique associations with other variables [2]. The plurality of constructs complicates theorizing and the development of psychology as a science [5]. When new constructs are being developed, it is therefore of great importance to carefully consider their uniqueness and distinctiveness in relation to existing constructs.

A frequently used scale for the measurement of mental wellbeing is the Warwick-Edinburgh Mental Wellbeing Scale (WEMWBS) [6]. At the time when the WEMWBS scales were developed, positive mental health was said to be under-researched, partly because of the lack of appropriate measures [7]. The original 14-items version was validated on community- and student samples in the UK. The scale was demonstrated to satisfy standard criteria for scale development [7]. It is now widely used in a number of countries and has been used in community- as well as clinical samples [8]. The Warwick-Edinburgh scales have also been widely used in evaluations of interventions to improve wellbeing [9].

Positive mental wellbeing as measured by the WEMWBS has, on the homepage of the Warwick Medical School, been described as located on one end of a continuum with the opposite end being mental illness, mental health problems, and psychiatric disorders. Because of this, the scale is considered to measure much more than the absence of mental illness [10]. Although, it is somewhat unclear what this implies from a psychometric point of view, it seems to suggest that the WEMWBS intends to measure something else than what is typically measured in screening instruments for mental illness. In fact, if the scale is truly located on the positive end of the mental health continuum, it should not really be able to measure mental health problems, similar to a thermometer limited to measure temperatures between 0 °C and 100 °C. Such an instrument would not be able to measure temperatures below 0 °C as all objects truly below 0 °C would simply read 0 °C. Measuring much more than the absence of mental illness could also align with the dual continuum model, which holds the assumption that mental illness and positive mental wellbeing are related but distinct dimensions [11].

Discriminant validity in the context of the WEMWBS has largely been studied by means of correlations with other measures [6, 12]. Although this may be a useful first step, it does not provide information on the extent to which the WEMWBS uniquely contributes to explain systematic reliable variance relative to existing instruments. In recent years, the bifactor model has become a popular framework to address this aspect of measurement redundancy and discriminant validity. It allows for the simultaneous modeling of shared and unique reliable variance, in other words to systematically distinguish between what scales have in common and what is unique to each scale. To our knowledge, only one study has thus far adopted such an approach in relation to the WEMWBS. In a large English general population sample, Böhnke and Croudace found considerable conceptual overlap between the General Health Questionnaire (GHQ), a measure of psychological distress, and the WEMWBS. They concluded that the items of the two scales mainly measure the same construct [3]. In the literature, this is typically referred to as essential unidimensionality, a situation in which a set of items or variables primarily measures a single underlying construct, even if small secondary influences or measurement errors exist [13].

In the present study we will compare the WEMWBS [6] with a widely used scale for measuring symptoms of depression, The nine-items version of the Patient Health Questionnaire (PHQ-9) [14]). PHQ-9 was originally developed as an instrument for screening for depression and tested in a large clinical sample [15]. Good psychometric properties of the PHQ-9 scale have been confirmed also in studies in other clinical samples [16, 17]. A Norwegian version of PHQ-9 was validated in a study among middle- and high school students. The scale was found to be uni-dimensional and to have good psychometric properties, including high internal consistency (Alpha = 0.88) [18]. In a more recent Norwegian study among a combined patient and non-patient sample, the internal consistency of PHQ-9 was shown to be excellent with alpha-values as high as 0.86 and 0.89 and with good psychometric properties for males as well as females [19].

The aim of our study was to examine the discriminant validity of the WEMWBS with respect to the PHQ-9 scale through a systematic examination of their intercorrelations, starting with simple correlations and progressing to bifactor modeling. We expect to find considerable overlap between these constructs, indicated by high correlations, which may suggest a lack of discriminant validity. Specifically, we hypothesize that the correlation between the WEMWBS and PHQ-9 will be moderately high, reflecting conceptual overlap while allowing for some uniqueness. Additionally, we anticipate that correlations between these scales modeled as latent variables will be even higher, potentially approaching standardized coefficients around −0.80. We expect the psychometric indices derived from the bifactor models to indicate that the combined items from both scales are essentially unidimensional. Furthermore, we anticipate that the correlations between the PHQ-9 and a set of demographic variables will mirror those of the WEMWBS but with reversed signs. Finally, we hypothesize that the associations between the WEMWBS scales and demographic variables will be significantly diminished when accounting for the shared reliable variance with the PHQ-9.

## Methods

### Participants

The results presented here are based on analyses of baseline data from an evaluation aimed at examining effects of a treatment for depression and anxiety; ‘Prompt Mental Health Care’ (PMHC), the Norwegian Version of Improving Access to Psychological Therapies [20]. PMHC is a municipal-based low-threshold mental health service for adults aged 18 years or older and includes both low-intensity (guided self-help, psychoeducational courses) and high-intensity (individual treatment) treatment forms of Cognitive Behavioural Therapy (CBT). PMHC uses variations of a “matched care” approach in which the treatment offered is based on a cooperative decision between client and therapist [20].

Eligibility for being offered the PMHC service is based on a defined set of inclusion and exclusion criteria. The main inclusion criterion was anxiety and/or mild to moderate depression (defined as Generalized Anxiety Disorder scale (GAD-7)/Patient Health Questionnaire (PHQ-9) scores above cutoff). The requirement of Norwegian language proficiency of participants was added to the trial for practical purposes.

The first 12 PMHC pilot sites were established in 2012–2013. The sites were distributed across several geographical areas in eastern, western, and central Norway, including both urban and rural areas. Nine of the pilot sites were located in individual municipalities, one through inter-municipal cooperation and two covered boroughs in the Oslo municipality. Data from one additional municipality were added to these data after recruitment of the first 12 pilot sites. Further details on the PMHC material are provided in a previous publication from this study [21].

The number of participants participating in the data collection at baseline was 1690. All participants provided written informed consent upon recruitment. Patients were either referred to the service by their general practitioners or they contacted the PMHC service themselves. Eligible patients were adults with anxiety and/or low to moderate levels of depression, and whose home address was within their respective PMHC site. Patients with suspected psychosis, bipolar disorder, personality disorder, severe drug abuse, or suicide risk were generally excluded from PMHC, and were referred to their general practitioner or specialized mental health care services.

### Measures

All measures included in this study were self-administered and were largely (> 95%) collected electronically.

#### WEMWBS

Two versions of the WEMWBS were used; the full version with fourteen items and the short version, which is based on a selection of seven out of the fourteen items. The original WEMWBS is a 14-item scale with response categories (Likert type) ranging from “none of the time” to “all of the time”. Some of the analyses in the present publication are based on a global sum score, or more precisely, a mean score, which was calculated by adding item scores and dividing by the number of items. This was done to preserve the scale range of individual items. It is assumed that the higher the global score, the higher the level of mental wellbeing. The original WEMWBS showed high reliability and low social desirability bias. Confirmatory factor analysis supported that the scale contains one single factor, but with a few correlated error terms [6]. The scale showed high positive correlations with other wellbeing scales, and low to moderate positive correlation with overall health [22]. Cronbach’s alpha was 0.91 in the current sample.

The short form of the scale, SWEMWBS, consist of 7 items and was found to have good psychometric properties as well [23, 24]. Haver and associates [25] assessed the validity of the SWEMWBS among Norwegian and Swedish hotel managers and reported acceptable psychometric properties. Cronbach’s alpha was 0.82 in the current sample.

Translation of the scale into Norwegian was carried out consistent with established standards and included forward- and back translations [12].

#### PHQ-9

The Patient Health Questionnaire (PHQ-9) was used to measure depressive symptoms [15]. It included 9 items based on each of the DSM-IV criteria for depression. Response categories ranged from 0 (“none of the time) to 3 (“all of the time”). Cronbach’s alpha was, in the current sample, 0.85. A mean score (sum score divided by number of items) was constructed and since it was a mean score, the range remained the same as for single items, 0 to 3.

#### Demographics

Gender, age, educational level (primary school, secondary school, higher education), relationship status (having a partner, not having a partner), immigration background (defined as being an immigrant or born in Norway with immigrant parents). Employment status was assessed by means of two questions, one multi-response item about employment status, and one multi-response item about sources of income [26]. Based on these two questions, participants were placed into four categories: 1) Employed, no social security benefits, 2) Employed, receives social security benefits, 3) Unemployed, receives social security benefits, and 4) Unemployed, receives no social security benefits.

### Statistical analyses

Valid meanscores (sumscores divided by number of items) for PHQ-9 and the WEMWBS scales were calculated for all cases which contained valid answers on at least half of the items. More than 95% of the sample had valid data on all items. Data were described with frequency and percentage distributions. Internal consistency of scales was estimated with Cronbach’s alpha. All this was done with SPSS version 28.0.1.1. For latent variable analyses, including bifactor models and MIMIC models, we used Mplus version 8 with the WLSMV estimator and Theta parameterization. The WLSMV estimator is tailored to the analysis of ordered categorical indicators and through a pairwise deletion of cases approach ensures good utilization of information in the relevant variables [27].

Simple bivariate correlations and the correlated 2-factor model provide initial insights into discriminant validity. However, comparing these correlations to arbitrary cutoffs can be misleading, especially when values are only slightly below the thresholds (e.g., 0.70 for manifest, 0.85 for latent correlations). In this context, the bifactor modeling approach offers a more refined, practically oriented method by separating shared factor variance from unique factor variance. This approach enhances the reliability and validity estimates of the constructs, making it easier to identify their true uniqueness. Additionally, bi-factor modeling derives a range of empirically embedded indices that quantitatively assess whether treating the two measures as a single construct would not result in substantial bias (essential unidimensionality). As such, this approach offers a more comprehensive understanding of the complex relationships between overlapping constructs [13, 28–30].

We estimated a bifactor model with one general factor that included all items from the PHQ-9 and WEMWBS (both the 7- and 14-item versions). In addition, we estimated one specific factor containing only the WEMWBS items. This is called a ‘Bifactor-(S-1) Model’ and has been described by Eid and associates [31]. In our context, the purpose of this procedure is to produce a well-defined general depressive symptoms factor which captures all reliable variance in the PHQ-9 items together with the part of the common WEMWBS variance that it shares with the PHQ-9 scale. In this way, the specific WEMWBS factor will consist of the reliable variance that is unique to the WEMWBS (orthogonal residual factor). Mplus syntax for estimation of the bifactor models are shown in Appendix to this publication.

The presence of correlated errors is a known issue for the WEMWBS [6, 12]. Some correlated error terms were included in the factor models to obtain acceptable levels of fit. There is evidence suggesting that including these correlated errors is not associated with substantial bias and does not represent well-defined separate factors [12]. As such, these correlated error terms should be interpreted as nuisance factors and reflect the reality that the constraints imposed by confirmatory factor models are often not fully met in practice. Moreover, when the number of correlated errors is small compared to the total number of correlations explained by the factor model, their overall impact is likely to be minimal. To verify this, we have also estimated our primary models without the inclusion of correlated errors. Criteria for inclusion of correlated errors were (i) improvement in fit measured with the model χ^2^-value using a figure-ground approach, (ii) standardized coefficients larger than 0.25, and (iii) overall fit of the model CFI larger than 0.95 and RMSEA values approaching 0.05 [32].

A number of psychometric indices were derived from the bifactor models: Global Omega (ω),Omega Hierarchical (ω_H_), Omega Subscale (ω_S_), Omega Hierarchical Subscale (ω_HS_), Explained Common Variance (ECV) and Percent of Uncontaminated Correlations (PUC) [29]. We also report a selection of other coefficients including Relative Omega, FD, H, and ARPB [28]. Estimation of the coefficients was carried out with Excel-based software produced by Dueber [33]. This tool does not fully account for the covariance explained by the correlated errors, but we expect this to have minimal impact as explained in the previous paragraph.

All the omega-coefficients are estimates of reliability similar to Cronbach’s alpha and vary from 0.00 to 1.00. But instead of being based on inter-item correlations, omega is based on common factoring models, is estimated from the factor loadings, and is most useful in the context of analysis of latent variables. Omega (global omega) is an estimate of reliability which includes the general factor as well as the specific factors combined. Omega S (subscale) includes subscale items only, but with their loadings on the general factor as well as the specific factor included in the calculations. Omega H (hierarchical) for the general factor is based on loadings on the general factor only. Omega HS (hierarchical specific) for specific factors is based on loadings on each subfactor separately without including loadings on the general factor. Relative Omega is Omega H divided by Omega and applies both to the general factor and to specific factors. For the general factor Relative Omega shows the proportion of the total reliable variance (general plus specific) that is covered by the general factor. For a specific factor, Relative Omega is the proportion of the reliable variance in the subscale that is independent of the general factor.

Explained Common Variance (ECV) for the general factor is the proportion of all common variance explained by that factor. For specific factors, in our context, ECV shows the strength of a specific factor relative to all explained variance only of the items loading on that specific factor [35]. This index is sometimes called ECV _NEW_ to distinguish this version from a different and older version of ECV. Percent Uncontaminated Correlations (PUC) represents the proportion of variance which only reflects variance from the general dimension [13].

FD is the correlation between factor scores and the factors. It is recommended that factor score estimates should only be used when FD > 0.90. H is a measure of construct replicability and represents the correlation between a factor and an optimally weighted item composite. High H values (H > 0.80) indicate a well-defined latent variable.

Average Relative Parameter Bias (ARPB) – an indicator of bias if items are forced into a unidimensional structure – is based on the difference between an item’s loading in the unidimensional solution and its general factor loading in the bifactor model, divided by the general factor loading in the bifactor model. An ARPB smaller than 10–15% is considered acceptable [30].

Different guidelines exist for assessing whether multidimensionality is severe enough to disqualify an instrument as primarily unidimensional. When ECV is above 0.80, relative bias will generally be lower than 5%, and when ECV is above 0.70 relative bias will generally be lower than 10%. Similar cut-offs can be applied to the PUC. However, when PUC values become lower, general ECV values are less important in predicting bias related to forcing a unidimensional model to multidimensional data. That is, when PUC values are lower than 0.80, general ECV values greater than 0.60 and ω_H_ > 0.70, the multidimensionality is not sufficiently large to reject the interpretation of the instrument as primarily unidimensional [13].

When presenting associations between latent variable outcomes and categorical (dichotomous) predictors in the MIMIC models, unstandardized coefficients are reported. This in order to ease interpretation. Since the latent outcome variables in these models all have a variance (and standard deviation) of 1.00, coefficients can be interpreted in terms of z-scores. In the first set of MIMIC models, each outcome was modelled as a single latent factor and regressed on the specified set of demographic variables (3 separate models: PHQ-9 as outcome, WEMWBS-14 as outcome, and WEMWBS-7 as outcome). In the second set of MIMIC models, the latent factors of the bifactor models were regressed on the same set of demographic variables (2 separate models: general factor and specific WEMWBS-14 factor, general factor and specific WEMWBS-7 factor).

## Results

### Descriptive and bivariate statistics

Table A1 (Appendix) shows percentage distributions on selected demographic variables. Three out of four study participants (74.8%) were women and three out of four were in the age range 21–50 years. The total age range was 18 to 86 years and mean age was 38.0 years (s.d. = 12.6). Almost nine out of ten have completed high school or higher levels of education and almost four out of ten were single. Three out of four had a job, more than half (55.7%) received no social security support. The proportion of immigrants in this study sample was 13.6%.

**Fig. 1:**
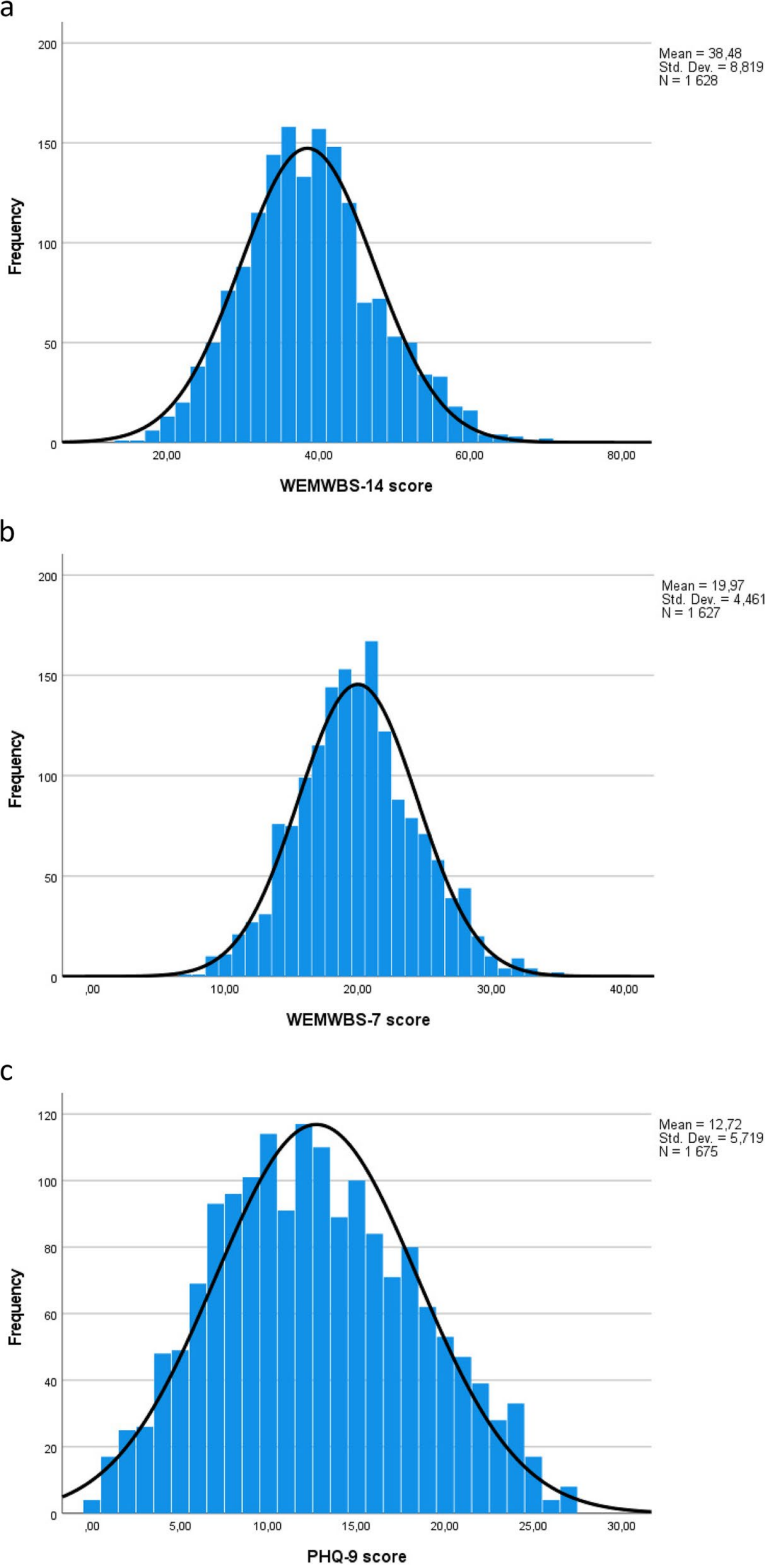
**a** Score distribution of the WEMWBS-14. **b** Score distribution of the WEMWBS-7. **c** Score distribution of the PHQ-9

Table A2 and Table A3 show percentage distributions on all single items of the PHQ-9 and the WEMWBS, respectively. The proportion of missing answers was low on both scales, varying from 0.9 to 1.1% on the PHQ-p scale and from 3.8 to 4.6% on the WEMWBS scale. Sum scores of PHQ-9, WEMWBS-14, and the WEMWBS-7 were normally distributed (see Fig. 1a and b for distribution of the WEMWBS-14 and WEMWBS-7). If the WEMWBS scales would only measure on one end of the mental health continuum, one would expect a clustering of participants with mental health problems on the lower end of the WEMWBS scales, in particular in this clinical sample, but this was not the case at all. The distribution of the PHQ-9 scores (see Fig. 1c) shows a similar distribution with no clustering of cases towards any of the ends of the scale and with only minor deviations from normality.

The correlation between meanscores (sumscores divided by number of items) for the two versions of the WEMWBS was 0.954. The correlations with PHQ-9 were −0.631 for the seven items version and −0.663 for the fourteen items. When modelled as latent variables, not allowing for any correlated error terms, the correlation of PHQ-9 with WEMWBS −7 was −0.772 and with WEMWBS −14, −0.795.

### Model fit of the bifactor models

**Fig. 2:**
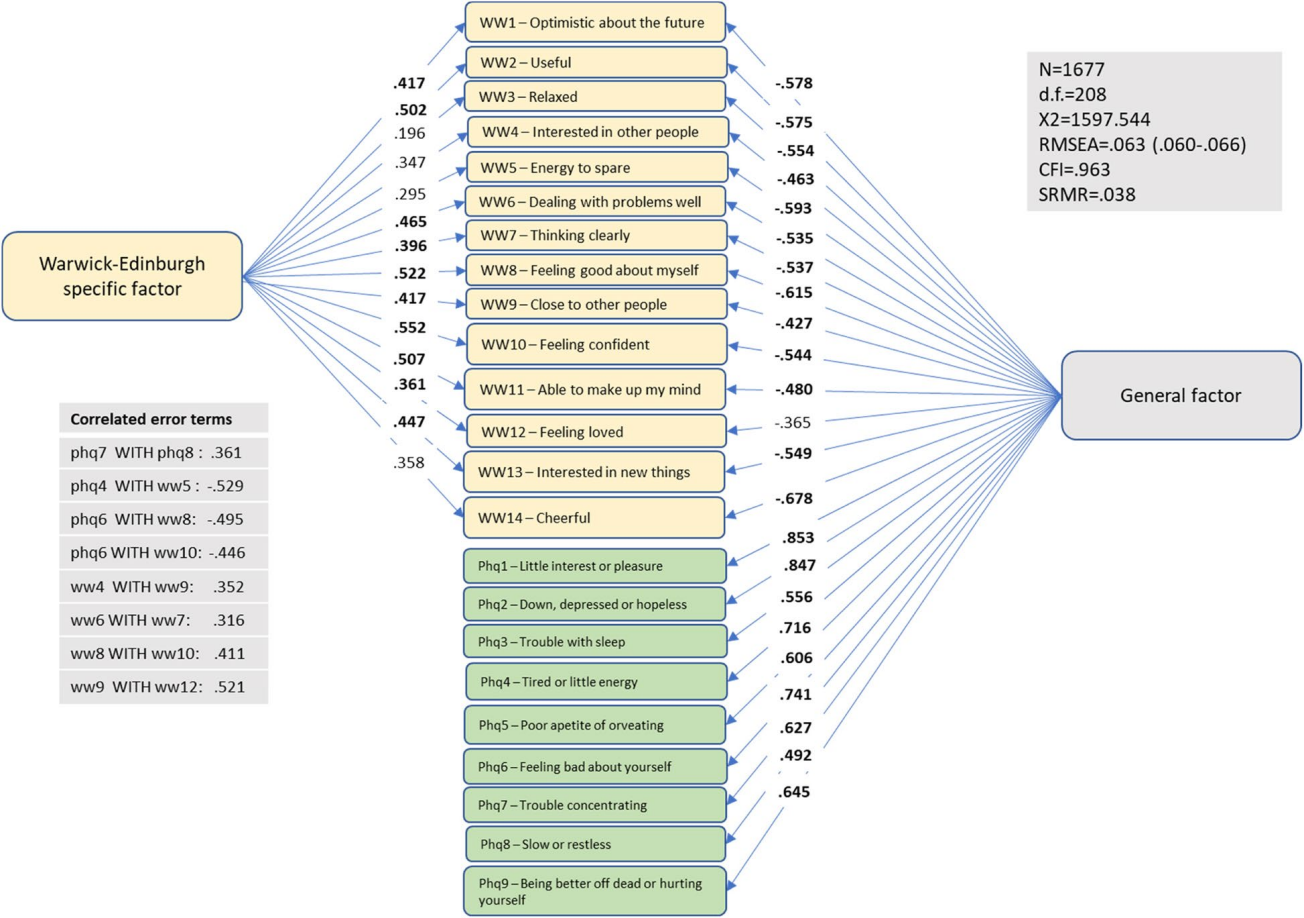
Patient Health Questionnaire (PHQ-9) and Warwick-Edinburgh Mental Wellbeing Scale (WEMWBS-14) – bifactor model 1

Figure 2 displays results from a bifactor model which, in addition to the PHQ-9 items, included all items from the WEMWBS-14. Eight correlated error terms had to be added to obtain adequate model fit. Twenty two out of the 23 items on the general factor, and 10 out of 14 on the specific WEMWBS factor had loadings higher than 0.40.

**Fig. 3:**
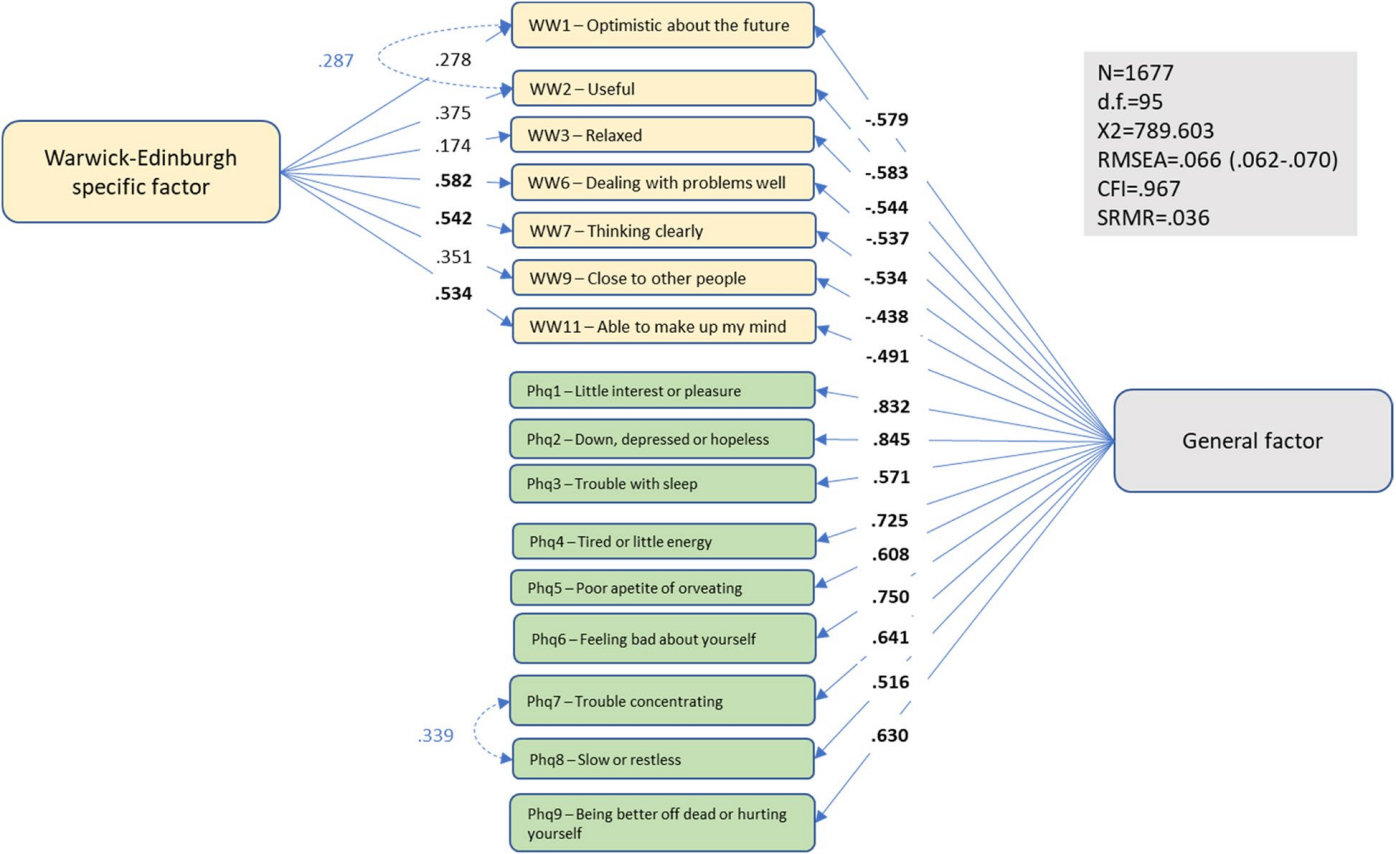
Patient Health Questionnaire (PHQ-9) and Warwick-Edinburgh Mental Wellbeing Scale (WEMWBS-7) – bifactor model 2

Figure 3 shows results from a similar bifactor analysis in which all items from the PHQ-9 and the seven-item version of the WEMWBS constituted the general factor. A separate, specific factor was defined for the WEMWBS items only. Only two, rather modest, correlated error terms had to be added to the model to obtain adequate fit (χ^2^ = 789.603; d.f. = 95; RMSEA = 0.066; CFI = 0.967). All sixteen loadings on the general factor were higher than 0.40. On the WEMWBS specific factor, three out of the seven loadings were higher than 0.40.

### Estimates of the bifactor model derived psychometric indices

**Fig. 4:**
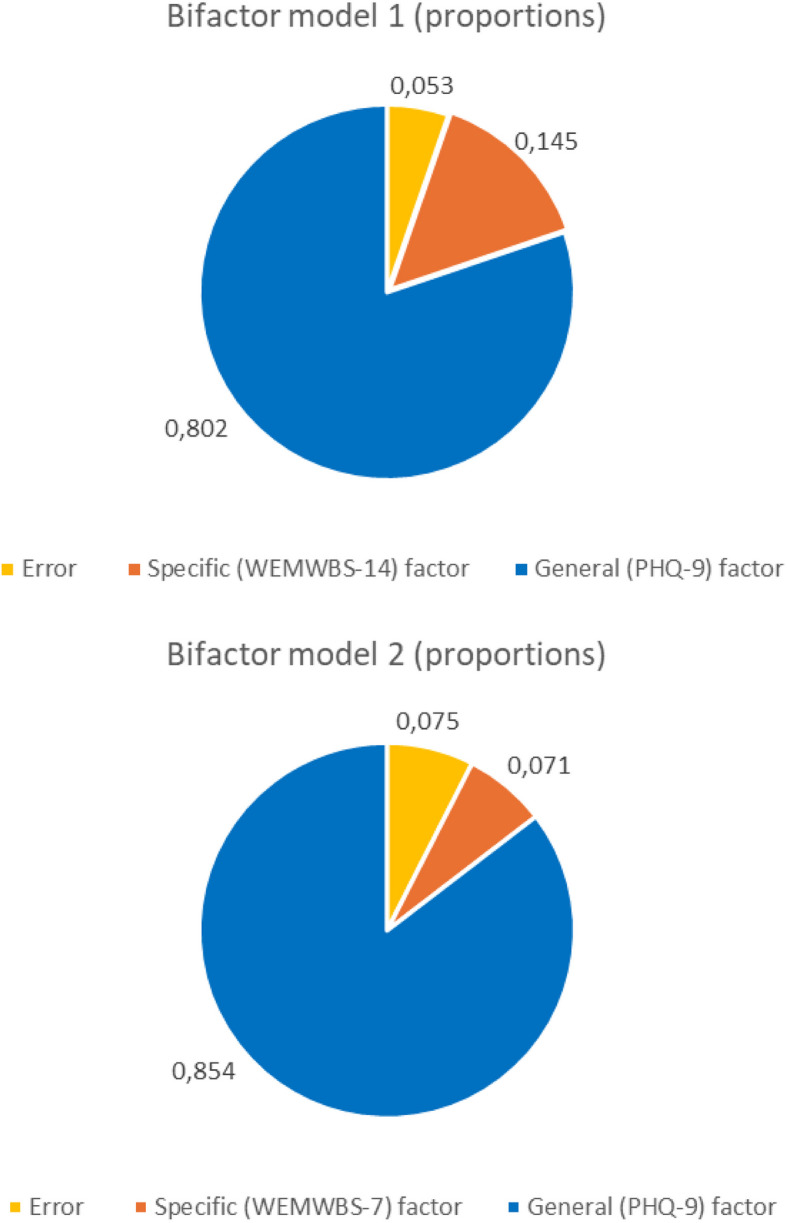
Variance components of bifactor models 1 and 2

In Table 1, Omega and other coefficients for the bifactor models are shown. Model 1 is based on PHQ-9 and WEMWBS-14, while Model 2 is based on the PHQ-9 and WEMWBS-7. The global omegas (ω) are 0.947 and 0.925 for Models I and II, respectively. This means that in both models, more than 90 percent of the variance for the all items combined can be attributed to the two factors; the general depressive symptoms factor and the one which includes Warwick-Edinburgh Mental Wellbeing Scale items only. Omega hierarchical (w_h_), the proportion of variance that can be attributed to the general factor, was 0.802 for Model 1 and 0.854 for model 2. This means that most of the reliable variance in total scores can be attributed to the general factor as indicated by a relative omega (w_H_/w) of 0.846 for Model 1 and 0.923 for Model 2. The variance partitioning is illustrated by the sector diagrams in Fig. 4 and visualize the dominance of the general PHQ-9 factor.

**Table 1:** Omega coefficients and related statistics for the bifactor models – with correlated error terms

		Model 1: PHQ-9 and WEMWBS-14	Model 2: PHQ-9 and WEMWBS-7
Complete set of items	Omega _Both factors_ (ω)	0.947	0.925
	Omega H _General factor_ (ω_H_)	0.802	0.854
	Relative Omega _General factor_	0.846	0.923
	ECV _General factor_	0.768	0.829
	H _General factor_	0,942	0.927
	FD _General factor_	0,961	0.958
WEMWBS items only	Omega S _Both factors_ (ω_S_)	0.924	0.854
	Omega HS _WEMWBS factor_ (ω_HS_)	0.345	0.315
	Relative Omega_WEMWBS factor_	0.373	0.369
	ECV _WEMWBS factor—new_	0,380	0.395
	H _WEMWBS factor_	0,762	0.636
	FD _WEMWBS factor_	0,871	0.827
% of uncontaminated correlations	PUC	0.640	0.825
Relative parameter bias	ARPB	0.177	0.106

The proportion of variance in the WEMWBS items explained by the two factors combined (ω_S_) was 0.924 in Model 1 and 0.854 in Model 2. The proportion of variance in the Warwick-Edinburgh Mental Wellbeing Scale items explained by the Warwick-Edinburgh factor (ω_hs_) was 0.345 (Model 1) and 0.315 (Model 2). This means that only moderate proportions of the reliable variance in WEMWBS scores can be attributed to the specific WEMWBS factors as indicated by a Relative Omega (ω_hs_/ω_S_) of 0.373 for Model 1 and 0.369 for Model 2.

The explained common variance (ECV) by the general factor was 0.768 for Model 1 and 0.829 for Model 2. The ECVs for the WEMWBS factor were 0.380 (Model 1) and 0.395 (Model 2). These results indicate that the general factors explained most of the extracted common variance while the WEMWBS factors explained much less, even when only estimated based on the WEMWBS items.

PUC was equal to.825 for Model 2 indicating that most correlations informed directly on the general factor. For model 1, PUC was 0.640. As explained in the methods section, when PUC is lower than 0.80, general ECV values greater than 0.60 and ω_H_ > 0.70, the multidimensionality is not sufficiently large to reject the interpretation of the instrument as primarily unidimensional. In our case, for Model 1, the ECV value was 0.768 and the ω_H_ was 0.802. The results for both models thus suggest that the instruments combined (PHQ-9 and each of the WEMWBS scales) are essentially unidimensional.

FD correlations for the general factors were 0.961 (Model 1) and 0.958 (Model 2). FD correlations for the specific factors were 0.871 (Model 1) and 0.827 (Model 2). As mentioned above, factor score estimates should only be used when FD > 0.90. H coefficients for the general factors were 0.942 (Model 1) and 0.927 (Model 2). H coefficients for the WEMWBS factor were 0.762 (Model 1) and 0.636 (Model 2). As previously mentioned, values higher than 0.80) indicate a well-defined latent variable.

The ARPB-value for model 2 is as low as 0.106. For model 1, which combines PHQ-9 with WEMWBS-14, the value is slightly higher (0.177) and slightly higher than what is considered acceptable.

The bifactor indices were re-calculated based on bifactor models without correlated error terms. Results indicated that the indices only changed marginally, confirming that the inclusion of these terms had negligible impact on the model estimates presented in Figs. 2 and 3.

### Associations of WEMWBS and PHQ-9 with other variables

Results from three separate MIMIC analyses are presented in Table 2 with latent scores of PHQ-9, WEMWBS-14 and WEMWBS-7 as dependent latent variables. PHQ-9 mean scores decreased with level of education and was higher among those who received social security benefits, among those who were single, and among those with immigrant background (all significant). Mean scores on the WEMWBS followed the same pattern, but with low scores in the groups where the PHQ-9 scores were high and high scores in the groups were the PHQ-9 scores were low.

**Table 2:** Unstandardized regression coefficients of associations between latent PHQ-9, WEMWBS-14 and WEMWBS-7 scores and selected demographic variables (MIMIC models)

		PHQ-9		WEMWBS-14		WEMWBS-7	
		Coefficients	Sign. (*p* <)	Coefficients	Sign. (*p* <)	Coefficients	Sign. (*p* <)
Gender	Female	.000	…	.000	…	.000	…
	Male	-.075	n.s	.007	n.s	-.020	n.s
Level of education	Primary	.201	.05	-.150	n.s	-.108	n.s
	High School	.000	…	.000	…	.000	
	University	-.164	.01	.125	.05	.142	.05
Job status	Employed, no social security benefits	.000	…	.000	…	.000	…
	Employed, receives social security benefits	.435	.001	-.354	.001	-.357	.001
	Unemployed, receives social security benefits	.206	.05	-.193	.05	-.272	.001
	Unemployed, receives no social security benefits	.026	n.s	.102	n.s	.013	n.s
Civil status	Not single	.000	…	.000	…	.000	…
	Single	.141	.05	-.156	.01	-.170	.01
Immigrant background	No	.000	…	.000	…	.000	…
	Yes	.463	.001	-.199	.01	-.206	.01
Age (unit 10 years)		-.056	.05	.059	.01	.086	.001
R^2^		.079		.046		.052	

Table 3 shows results of two MIMIC bifactor analyses. The associations between the general factor (reflecting PHQ-9) and the demographic predictors are largely the same as for the models presented in Table 2. Of particular interest in our context are the results for the specific factors, WEMWBS-14 in Model 1 and WEMWBS-7 in model 2. WEMWBS-14 had lost most of its associations with demographic predictors, with relationship status and immigrant background being the only exceptions. The association between relationship status and the WEMWBS-14 was slightly weaker than the one seen for the model presented in Table 2. The association between Immigrant background and the WEMWBS-14 changed direction when compared with the model presented in Table 2. In the simple MIMIC model, those who had an immigrant background had a lower level of mental wellbeing (−0.206). When adjusting for the general (PHQ-9) factor as shown in Table 3, those who had an immigrant background had a higher score (0.217). As the residual WEMWBS factor was not well-defined (H-index < 0.80), a substantial interpretation of this result may not be warranted. The WEMWBS-7 had also lost most of its associations with demographic predictors, age being the only exception.

**Table 3:** Unstandardized regression coefficients of the associations between the bifactor (S-1) model factors and selected demographic variables (MIMIC model)

		Model 1				Model 2			
		General (PHQ-9)		WEMWBS-14		General (PHQ-9)		WEMWBS-7	
		Coefficients	Sign. (*p*<)	Coefficients	Sign. (*p*<)	Coefficients	Sign. (*s*<)	Coefficients	Sign (*p*<)
Gender	Female	.000	…	.000	…	.000	…	.000	…
	Male	-.081	n.s.	-.097	n.s.	-.065	n.s.	-.095	n.s.
Level of education	Primary	.196	.05	.000	n.s.	.198	.05	.074	n.s.
	High School	.000	…	.000	….	.000	…	.000	…
	University	-.144	.05	.036	n.s.	-.155	.01	.044	n.s.
Job status	Employed, no social security benefits	.000	…	.000	…	.000	…	.000	…
	Employed, receives social security benefits	.435	.001	-.015	n.s.	.427	.001	-.026	n.s.
	Unemployed, receives social security benefits	.193	.05	-.075	n.s.	.205	.01	-.165	n.s.
	Unemployed, receives no social security benefits	.031	n.s.	.201	n.s.	.042	n.s.	.102	n.s.
Civil status	Not single	.000	…	.000	…	.000	…	.000	…
	Single	.109	n.s.	-.136	.05	.153	.01	-.048	n.s.
Immigrant background	No	.000	…	.000	…	.000	…	.000	…
	Yes	.442	.001	.217	.05	.426	.001	.145	n.s.
Age (unit 10 years)		-.051	.05	.035	n.s.	-.052	.05	.082	.01
R^2^		.073	.001	.018	.05	.073	.001	.020	.05

When examining the relationship between outcomes and demographic factors, it is interesting to note that the multiple R squared was higher for PHQ-9 than for the WEMWBS. It is also worth noticing that the multiple R squared were particularly low when the WEMWBS were outcomes after adjusting for the general factor (PHQ-9).

## Discussion

The overall aim of our study was to examine the discriminant validity of the WEMWBS with reference to an existing and commonly used measure of depressive symptoms (the Patient Health Questionnaire).

The most important findings can be summarized as follows:

(i) The correlations between PHQ-9 and the WEMWBS scales were strong and negative, as high as 0.80 in the latent model analyses with the full (14 items) WEMWBS scale.
(ii) Practically all psychometric indices derived from the bifactor models suggested that the WEMWBS-7 and PHQ-9 combined, and the WEMWBS-14 and PHQ-9 combined were essentially unidimensional.
(iii) The associations between PHQ-9 and a set of demographic variables were similar to associations between the WEMWBS scales and the same set of demographic variables, only with reversed signs.
(iv) Associations between the residual WEMWBS scales and a set of demographic variables generally faded away, and in one case, changed direction, when removing the reliable variance accounted for by the general depressive symptoms factor.

Our results confirm that relying solely on the size of manifest correlations between the WEMWBS scales and the PHQ-9 scale (−0.63 for WEMWBS-7 and −0.66 for WEMWBS-14) can lead to misleading conclusions about an instrument's discriminant validity, as these correlations were below the typical 0.70 threshold. The same holds true for latent factor correlations based on the standard 2-factor model. Supplementary evidence from the bifactor models indicated that this conclusion is not warranted in the case of the WEMWBS.

Altogether, our study suggests that the WEMWBS scales may lack discriminant validity with reference to symptoms of depression as measured by PHQ-9 in a sample of patients with mild to moderate anxiety and/or depression. This is largely in line with the study by Böhnke and Croudace, which showed that the WEMWBS was not much different from measuring psychological distress, which itself is mainly driven by symptoms of depression [3]. It may therefore not be justified to present the WEMWBS as measuring a new concept. The WEMWBS may essentially be measuring an already existing concept, but with a different label and scores defined in the opposite direction, a situation known as the jangle fallacy (i.e. the mistake of assuming that two things are different because they have different names, even though they actually measure the same or very similar concepts) [35].

The results of our study suggest that the WEMWBS may neither represent one end of the mental health continuum, nor a dual continuum model in which wellbeing and symptoms of depression are two related but distinct dimensions. A bipolar conceptualization of mental health seems to fit better with present and earlier findings [3], which suggests that a person scoring low on the WEMWBS has poor mental health, and a person scoring high has good mental health. This would be in line with the interpretation of the PHQ-9, just in the opposite direction. That is, a person scoring low on the PHQ-9 has good mental health and a person scoring high has poor mental health. As such, the WEMWBS may be redundant in the presence of the PHQ-9.

### Strengths and limitations

The present study involved a relatively large sample of participants, and the instruments used were well-tested and appropriately adapted to both the Norwegian language and context. Bifactor and MIMIC modeling are highly effective statistical tools that aligned well with the aims of the study. Since the sample included only primary health care patients with mild to moderate depression and anxiety, the PHQ-9 and WEMWBS items may show more variation and less skewness. This may differ from what would be expected in a random general population sample, in particular for PHQ-9. This is supported by the distributions shown in Figs. 1a-1c.

The fact that the sample comprised patients only, is also a limitation of this study. Findings may not straightforwardly be generalized to the general adult Norwegian population. However, Böhnke and Croudace came to the same conclusion regarding the discriminant validity of the WEMWBS based on data from a general population study in England [3]. Another limitation of this study is the relatively small number of demographic variables available for being included in our models. A broader selection would have strengthened the validity and generalizability of our findings. Finally, the present findings can also not be generalized to other instruments measuring mental wellbeing.

To enhance the generalizability of our findings, future studies should include diverse samples from both the general population and specific patient groups, across various countries and cultures. This would help determine whether our results hold true in broader contexts. It would also be of great interest to adopt a bifactor model approach to examine the discriminant validity of related mental wellbeing questionnaires, such as the PANAS scale [35], Satisfaction with Life Scale [35], Scale of Psychological Wellbeing [35], the Short Depression-Happiness Scale [35], and the WHO Wellbeing Index [35]. In this regard, it’s important to include a validated measure of depressive symptoms, for example the PHQ-9. Finally, identifying aspects of mental wellbeing that are sufficiently distinct from what is measured by scales like PHQ-9 would also be a relevant avenue for future research. Based on the large, correlated error between WEMWBS items 9 and 12, aspects associated with social relationships could be a potential candidate in this context (see Fig. 2).

## Conclusions

The present study provides evidence that both the short and full versions of the WEMWBS scales, when combined with the PHQ-9 into joint scales, are essentially unidimensional. The associations between the WEMWBS scales and selected correlates mirror those between the PHQ-9 and the same set of correlates. Based on our sample of primary care patients with mild-to-moderate anxiety and/or depression, we conclude that the WEMWBS may lack discriminant validity in relation to the PHQ-9. This raises significant concerns about the validity of the WEMWBS as a measure of positive mental wellbeing. Future research, particularly in community samples, is needed to confirm the generalizability of these findings and to determine whether revisions to the WEMWBS may be necessary.

## Supplementary Information

Supplementary Material 1.

## Data Availability

The datasets analyzed during the current study are not publicly available due to ethical restrictions and personal data protection but are available from the third author (ORS) on reasonable request.

## Abbreviations

ARPB: Average Relative Parameter Bias
CBT: Cognitive Behavioural Therapy
CFI: Comparative Fit Index
ECV: Explained Common Variance
FD: Factor Determinacy
H: A measure of construct replicability
IAPT: Improving Access to Psychological Therapies
MIMIC: Multiple Indicators Multiple Causes – used in the context of structural equation modeling.
PHQ-9: The nine-items version of the Patient Health Questionnaire
PMHC: Prompt Mental Health Care – A Norwegian low-threshold psychological therapy programme similar to the British Improved Access to Psychological Therapies (IAPT) services
PUC: Percent of Uncontaminated Correlations
RMSEA: Root Mean Square Error of Approximation
SRMR: Standardized Root Mean Square Residual
WEMWBS: The Warwick-Edinburgh Mental Wellbeing Scale
WLSMV: Weighted least square mean- and variance-adjusted estimator
ω: Omega – a family of reliability coefficients for latent variables
