# Supplementary material for: Positive mental wellbeing or symptoms of depression? Discriminant validity of the Warwick-Edinburgh Mental Wellbeing Scale
Source: BMC Psychiatry. 2025 May 15;25:487. doi: 10.1186/s12888-025-06922-0 (PMC12080168; doi:10.1186/s12888-025-06922-0)
Supplement: Supplementary file 1 — Supplementary Material 1. [file 12888_2025_6922_MOESM1_ESM.docx]

APPENDIX

Table A1: Study sample demographics

| Variable | Categories | n | % |  |
| --- | --- | --- | --- | --- |
| Gender | Men | 420 | 25.2 |  |
|  | Women | 1247 | 74.8 |  |
|  | Missing | 23 | --- |  |
| Age | 18-20 | 107 | 6.4 |  |
|  | 21-30 | 442 | 26.5 |  |
|  | 31-40 | 446 | 26.7 |  |
|  | 41-50 | 380 | 22.8 |  |
|  | 51-60 | 211 | 12.6 |  |
|  | 61-70 | 84 | 5.0 |  |
|  | Missing | 20 | --- |  |
| Highest completed education | Primary or secondary school | 178 | 10.7 |  |
|  | High School | 753 | 45.3 |  |
|  | University or university college | 732 | 44.0 |  |
|  | Missing | 27 | --- |  |
| Marital status | Not single | 1016 | 61.1 |  |
|  | Single | 648 | 38.9 |  |
|  | Missing | 26 | --- |  |
| Job status | Job, no support | 642 | 38,4 |  |
|  | Job, receives support | 614 | 36,7 |  |
|  | Unemployed, no support | 290 | 17,3 |  |
|  | Unemployed, receives support | 127 | 7,6 |  |
|  | Missing | 17 | --- |  |
| Immigration status | Native Norwegian | 1436 | 86.4 |  |
|  | Immigrant | 226 | 13.6 |  |
|  | Missing | 28 | --- |  |
| Total |  | 1690 | 100.0 |  |

Table A2: Patient Health Questionnaire (PHQ-9): Single item percentage distributions

|  | Not at all | Some days | More than half of the days | Almost every day | Total | |
| --- | --- | --- | --- | --- | --- | --- |
|  | % | % | % | % | % | N |
| 1. Little interest or pleasure in doing things | 9.3 | 45.3 | 25.0 | 20.4 | 100.0 | 1671 |
| 2. Feeling down, depressed, or hopeless | 6.8 | 42.5 | 26.5 | 24.3 | 100.0 | 1674 |
| 3. Trouble falling or staying asleep, or sleeping too much | 13.6 | 31.4 | 23.2 | 31.0 | 100.0 | 1674 |
| 4. Feeling tired or having little energy | 3,2 | 27.8 | 30.7 | 38.3 | 100.0 | 1675 |
| 5. Poor appetite or overeating | 22.4 | 36.0 | 20.6 | 21.0 | 100.0 | 1673 |
| 6. Feeling bad about yourself or that you are a failure or  have let yourself or your family down | 10.6 | 34.4 | 27.9 | 27.1 | 100.0 | 1672 |
| 7. Trouble concentrating on things, such as reading the  newspaper or watching television | 21.9 | 37.8 | 20.6 | 19.8 | 100.0 | 1674 |
| 8. Moving or speaking so slowly that other people could  have noticed. Or the opposite being so fidgety or  restless that you have been moving around a lot more  than usual | 47.6 | 33.0 | 12.3 | 7.1 | 100.0 | 1671 |
| 9. Thoughts that you would be better off dead, or of  hurting yourself | 67.7 | 25.7 | 4.2 | 2.3 | 100.0 | 1674 |

Table A3: Warwick Edinburgh Mental Wellbeing Scale (WEMWBS): Single item percentage distributions

|  | Not at all | Seldom | Parts of the time | Often | All the time | Total | |
| --- | --- | --- | --- | --- | --- | --- | --- |
|  | % | % | % | % | % | % | N |
| 1. I’ve been feeling optimistic about the future | 8.6 | 31.0 | 40.2 | 18.1 | 2.2 | 100.0 | 1612 |
| 2. I’ve been feeling useful | 7.3 | 32.0 | 40.5 | 18.1 | 2.1 | 100.0 | 1617 |
| 3. I’ve been feeling relaxed | 11.1 | 43.0 | 35.6 | 9.2 | 1.1 | 100.0 | 1613 |
| 4. I’ve been feeling interested in other people | 4.0 | 21.6 | 39.0 | 28.2 | 7.2 | 100.0 | 1617 |
| 5. I’ve had energy to spare | 27.8 | 44.2 | 21.7 | 5.5 | 0.8 | 100.0 | 1624 |
| 6. I’ve been dealing with problems well | 7.4 | 28.3 | 47.8 | 14.9 | 1.5 | 100.0 | 1618 |
| 7. I’ve been thinking clearly | 4.3 | 20.2 | 47.0 | 23.1 | 5.4 | 100.0 | 1622 |
| 8. I’ve been feeling good about myself | 16.7 | 38.1 | 33.1 | 10.7 | 1.4 | 100.0 | 1622 |
| 9. I’ve been feeling close to other people | 6.2 | 23.8 | 35.8 | 27.8 | 6.4 | 100.0 | 1623 |
| 10. I’ve been feeling confident | 19.5 | 40.6 | 28.4 | 9.7 | 1.8 | 100.0 | 1623 |
| 11. I’ve been able to make up my own mind about things | 3.6 | 19.2 | 43.0 | 25.9 | 8.3 | 100.0 | 1624 |
| 12. I’ve been feeling loved | 7.9 | 19.1 | 29.7 | 27.9 | 15.4 | 100.0 | 1626 |
| 13. I’ve been interested in new things | 17.1 | 36.9 | 29.0 | 13.7 | 3.3 | 100.0 | 1616 |
| 14. I’ve been feeling cheerful | 3.3 | 28.3 | 52.5 | 14.4 | 1.6 | 100.0 | 1620 |

**Mplus syntax for the bifactor model presented in Figure 2 (PHQ-9 and WEMWBS-14).**

!GEN – general factor

!WEM – specific Warwick-Edinburgh scale factor

!ww - Warwick Edinburgh scale items

!phq – PHQ-9 items

CATEGORICAL ARE phq phq2 phq3 phq4 phq5 phq6

phq7 phq8 phq9 ww1 ww2 ww3 ww4 ww5 ww6 ww7

ww8 ww9 ww10 ww11 ww12 ww13 ww14;

ANALYSIS:

ESTIMATOR=WLSMV;

PARAMETERIZATION=THETA;

MODEL:

GEN BY phq1*

phq2 phq3 phq4 phq5 phq6 phq7 phq8 phq9 ww1

ww2 ww3 ww4 ww5 ww6 ww7 ww8 ww9 ww10 ww11

ww12 ww13 ww14;

WEM BY ww1*

ww2 ww3 ww4 ww5 ww6 ww7 ww8 ww9 ww10 ww11

ww12 ww13 ww14;

GEN WITH WEM@0;

GEN@1;

WEM@1;

!Error correlations

phq7 WITH phq8; phq4 WITH ww5; phq6 WITH ww8;

phq6 WITH ww10; ww4 WITH ww9; ww6 WITH ww7;

ww8 WITH ww10; ww9 WITH ww12;

**Mplus syntax for the bifactor model presented in Figure 3 (PHQ-9 and WEMWBS-7).**

!GEN – general factor

!WEM – specific Warwick-Edinburgh scale factor

!ww - Warwick Edinburgh scale items

!phq – PHQ-9 items

CATEGORICAL ARE phq1 phq2 phq3 phq4 phq5 phq6 phq7

phq8 phq9 ww1 ww2 ww3 ww6 ww7 ww9 ww11;

ANALYSIS:

ESTIMATOR=WLSMV;

PARAMETERIZATION=THETA;

MODEL:

GEN BY phq1*

phq2 phq3 phq4 phq5 phq6 phq7 phq8 phq9

ww1 ww2 ww3 ww6 ww7 ww9 ww11;

WEM BY ww1*

ww2 ww3 ww6 ww7 ww9 ww11;

GEN WITH WEM@0;

GEN@1;

WEM@1;

!Error correlations

Phq7 WITH phq8;

Ww1 WITH ww2;
